# Exploratory activity-based direct cost analysis of robotic platforms in urologic surgery

**DOI:** 10.1007/s11701-026-03596-y

**Published:** 2026-06-19

**Authors:** Paola Picozzi, Umberto Nocco, Eleonora Bossi, Daniela Gattuso, Chiara Labate, Greta Puleo, Veronica Cimolin

**Affiliations:** 1 Department of Electronics, Information and Bioengineering, Politecnico di Milano, Milano, 20133 Italy; 2 Clinical Engineering Department of ASST Grande Ospedale Metropolitano Niguarda, Milano, 20162 Italy; 3 Operation Management Department, ASST Grande Ospedale Metropolitano Niguarda, Milano, 20162 Italy; 4 Department of Public Health, Experimental and Forensic Medicine, University of Pavia, Pavia, 27100 Italy; 5 Istituto Auxologico Italiano, IRCCS, S. Giuseppe Hospital, Piancavallo, Oggebbio, 28824 Verbania Italy

**Keywords:** Robotic surgery, New robotic surgical platforms, Cost-analysis, Urology

## Abstract

Robotic-assisted surgery has become increasingly widespread across surgical specialties over the last two decades. However, the high costs associated with robotic systems remain a major limitation to their diffusion. Recently introduced robotic platforms aim to increase market competition and potentially reduce procedural costs. This study evaluated the direct in-hospital costs associated with three robotic platforms in urologic surgery. A retrospective single-center cost analysis was conducted on robotic-assisted procedures performed in 2023. A total of 284 robotic-assisted radical prostatectomies (RALP) were included (200 Da Vinci, 77 Hugo™ RAS, and 7 Versius^®^), while 77 robotic-assisted partial nephrectomies were analyzed (57 Da Vinci and 20 Hugo™ RAS). Partial nephrectomy analysis therefore involved only two platforms. Cost estimation was performed using an activity-based costing approach from the hospital/provider perspective, including direct costs related to robotic equipment, maintenance, consumables, operating room occupancy, personnel, and hospitalization. For partial nephrectomy, no statistically significant difference in total direct cost was observed between Da Vinci (€7856.89 ± 1335.76) and Hugo™ (€8226.36 ± 1481.85) platforms (*p* = 0.16), although operating room time and related costs were significantly higher with Hugo™ (*p* = 0.03 and *p* = 0.04, respectively). For RALP, mean total costs were €8077.99 (Da Vinci), €7867.92 (Hugo™), and €8430.76 (Versius^®^), without statistically significant differences (*p* = 0.13). Operative and operating room times were significantly longer with Versius^®^ (*p* < 0.05). Due to the limited number of Versius^®^ procedures, findings involving this platform should be considered exploratory. In this single-center experience, direct in-hospital costs associated with robotic-assisted urologic procedures appeared broadly comparable across platforms. However, findings should be interpreted cautiously given the retrospective design, unbalanced cohorts, absence of detailed case-mix adjustment, and the limited Versius^®^ sample size.

## Introduction

Over the past two decades, robotic-assisted surgery has undergone a remarkable transformation, evolving from a niche innovation to a widely adopted tool across numerous surgical specialties worldwide [1, 2]. Its growth has been driven by continuous technological advancements, improved training protocols, and a growing body of evidence supporting its clinical efficacy. One of the most significant milestones in this field was the introduction of the Da Vinci Surgical System, developed by Intuitive Surgical, which was among the first robotic platforms to be successfully implemented in clinical practice in the year 2000 [3].

Thanks to its early market entry and a comprehensive and strategically constructed portfolio of patents, the Da Vinci system was able to maintain an exclusive position in the global market for nearly two decades [4, 5]. These patents, covering both mechanical and software components, effectively blocked the emergence of any meaningful competitors, allowing Intuitive Surgical to establish a virtual monopoly in the field of robotic surgery. This monopoly, while driving innovation and widespread adoption in certain settings, also had notable downsides: it enabled the company to set extremely high prices for both the initial acquisition of the system and its ongoing maintenance and consumables. As a consequence, many healthcare institutions—particularly those in low- and middle-income countries or with limited budgets—were excluded from access to robotic surgical technology, leading to inequities in healthcare delivery and limiting the potential global impact of this innovation.

Nevertheless, despite the substantial economic barrier to entry, robotic-assisted surgery has consistently demonstrated its ability to improve the quality, precision, and overall outcomes of surgical procedures, not only for patients but also for the surgical team [6, 7]. These benefits, including reduced blood loss, shorter hospital stays, and improved ergonomics for surgeons, have been well-documented and widely endorsed by the surgical community. Fueled by this strong clinical validation and increasing demand, numerous medical technology companies began investing in the development of alternative robotic platforms, particularly in anticipation of the expiration of Intuitive Surgical’s most critical patents.

In recent years, following the lapse of these protections, a new wave of competition has entered the robotic surgery market [8, 9]. Among the most notable and advanced of these new systems are the Hugo™ Robotic-Assisted Surgery (RAS) System, developed by Medtronic, and the Versius^®^ Surgical Robotic System, introduced by CMR Surgical. These platforms aim not only to replicate the clinical performance of Da Vinci but also to offer more flexible, cost-effective, and modular solutions, which are easier to integrate into existing surgical workflows and potentially more accessible to a wider range of healthcare providers. Their introduction has sparked a new phase of positive competition, fostering technological innovation, increased market dynamism, and, crucially, a potential reduction in costs associated with robotic surgical technologies [4, 8, 9].

While there is a growing number of studies assessing and comparing the clinical effectiveness of these new robotic systems in various surgical domains [10–15], it is important to note that, to the best of the authors’ knowledge, only one published study has conducted a direct economic comparison between the Da Vinci and Hugo™ systems [16]. This indicates a clear and significant gap in the literature, particularly concerning the economic implications of the adoption of emerging robotic platforms, which is a crucial factor for hospitals and healthcare systems when making procurement and investment decisions.

Recent studies on Hugo™ RAS adoption in urologic surgery have reported encouraging perioperative and functional outcomes, supporting the feasibility and safety of the platform for both radical prostatectomy and partial nephrectomy procedures. In particular, emerging evidence suggests that the Hugo™ system may achieve perioperative outcomes comparable to established robotic platforms, although additional high-quality comparative data remain necessary [17–20].

This study seeks to address that gap by presenting a comprehensive comparative economic analysis of the Da Vinci, Hugo™, and Versius^®^ robotic platforms, specifically within the field of urologic surgery. The analysis focuses on two of the most commonly performed procedures in this specialty—partial nephrectomy and radical prostatectomy—and evaluates a range of direct costs, including system acquisition, maintenance contracts, disposable instruments, operative time, and length of stay. By providing a clear and detailed comparison, this study aims to contribute to more informed decision-making regarding the implementation of robotic systems in surgical practice.

## Methods

This is a cost analysis study that aims to evaluate and compare the economic impact of robotic-assisted procedures performed using Da Vinci Surgical System (Intuitive Surgical), Hugo™ RAS System (Medtronic), and Versius^®^ Surgical System (CMR Surgical). The study was conducted at ASST Grande Ospedale Metropolitano Niguarda, a public referral centre for robotic surgery located in Milan, Italy. Data were collected and analysed retrospectively from the year 2023, during which the centre was equipped with two Da Vinci Xi systems, one Hugo™ RAS, and one Versius^®^ system.

### Procedure selection

In order to ensure a robust comparison across the three robotic platforms, urologic procedures were selected based on two criteria: (1) Procedures carried out using at least two robotic platforms, so as to enable a comparative analysis and (2) a minimum of five procedures performed per platform. These two criteria were defined to have the possibility to compare the surgical procedures’ outcomes across all the different platforms. Based on these criteria, selected because they have been performed using more than one robotic platform: radical prostatectomy and partial nephrectomy. These are also the most commonly performed robotic procedures in urology and offer a degree of standardization in the use of robotic kits at our institution, allowing for more accurate cost estimation.

**Fig. 1:**
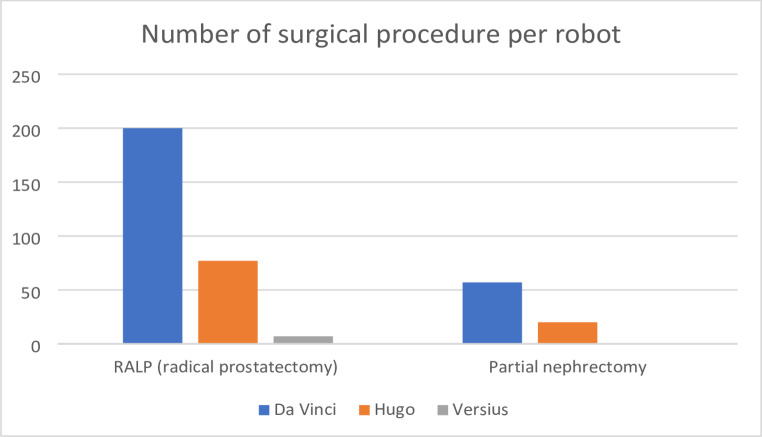
Number of surgical procedures per robot

In total, 284 robotic-assisted radical prostatectomies (RALPs) were analysed (Fig. 1): 200 performed with the Da Vinci systems, 77 with Hugo™, and 7 with Versius^®^. For partial nephrectomies, 77 cases were included: 57 performed with Da Vinci and 20 with Hugo™. All data refer to operations conducted in 2023. The number of procedures performed with the Versius^®^ platform was limited because its adoption in the urology department occurred later during the study period compared to the Da Vinci and Hugo™ systems. For Hugo™ and Versius^®^ systems, only cases performed beyond the initial learning curve were included. Furthermore, all participating surgeons received dedicated training on each robotic platform, and cases where trainees acted as primary console surgeons were excluded to reduce variability in operative times and outcomes.

### Cost analysis

The primary objective of this study was to evaluate and compare the direct costs associated with robotic-assisted radical prostatectomy (RALP) and partial nephrectomy performed using the Da Vinci, Hugo™ RAS, and Versius^®^ platforms. To achieve this, the analysis considered three main economic endpoints: the cost of the operation, the cost of the hospital stay, and the total cost, defined as the sum of the first two.

The analysis was conducted from the provider perspective and included direct in-hospital costs only. This study was not designed as a cost-effectiveness analysis because clinical effectiveness endpoints and long-term outcomes were not evaluated.

A micro-costing strategy was adopted, based on an Activity-Based Costing (ABC) approach [21], to estimate the direct costs of each robotic procedure. This methodology allowed for a detailed allocation of resources by linking specific costs to individual clinical activities. Equipment costs (including system rental, maintenance, and robotic kits), operating room occupation (personnel and facility costs), and hospitalization expenses were each attributed based on actual usage and procedure duration. Data were derived from the regional procurement framework (ARIA Lombardia 2022), the hospital’s surgical management system, and financial records. This level of cost granularity enables a more accurate comparison between the robotic platforms under investigation, in real-world clinical settings.

The calculation of the operation cost was based on the aggregation of multiple components. Firstly, equipment-related expenses were included, which comprised the annual robot rental fee, maintenance costs, and the per-procedure cost of the robotic instrument kit. These data were derived from the 2022 ARIA regional tender conducted in Lombardy, which defined a per-procedure price for each robotic platform. In accordance with regional guidelines, the maintenance cost was prorated assuming a minimum of 250 procedures per robot per year. A one-way sensitivity analysis was performed to assess the impact of different annual procedural volumes on total direct costs. Since robotic equipment costs are strongly dependent on annual utilization, the per-procedure equipment cost was recalculated assuming annual volumes of 150, 200, 250, 300, and 400 procedures per platform. Other cost components, including robotic kits, operating room costs, personnel costs, and hospitalization costs, were kept constant. The resulting impact on total direct procedural costs was assessed separately for radical prostatectomy and partial nephrectomy. The robotic kit refers to the standardized set of consumable instruments necessary to perform each surgery, typically consisting of four robotic arms and other platform-specific tools.

Secondly, personnel costs were calculated by accounting for all clinical and support staff involved in the procedure, including surgeons, anaesthesiologists, nurses, and operating room assistants. Using time-based data from the hospital’s OR management system, the cost for each staff category was determined by multiplying the per-minute cost by the recorded operative time of each procedure.

Finally, the cost of operating room usage was included, encompassing all expenses related to OR occupancy. This included the use of pharmaceuticals, medical gases such as oxygen and carbon dioxide, electricity, and other overhead resources. A standardized cost-per-minute for the operating room was applied and multiplied by the duration of each surgical procedure.

The hospital stay cost was assessed by multiplying the number of inpatient days by the daily hospitalization cost, which includes medical care, nursing services, accommodation, and facility use. The total cost per procedure was then determined by summing the calculated operation cost and the corresponding hospital stay cost, providing a comprehensive estimate of the direct expenditure for each robotic platform.

### Statistical analysis

Statistical analysis was carried out using Python 3.10. Data processing and organization were managed through the use of the pandas and numpy libraries, while statistical tests for both parametric and non-parametric variables were performed using scipy.stats. For visual inspection of data distribution, matplotlib.pyplot and seaborn were employed to produce histograms and Q-Q plots. In cases where post hoc analysis was necessary, the statsmodels library was used.

The normality of data distribution was assessed through both the Shapiro-Wilk test and graphical evaluation. Continuous variables were presented either as mean and standard deviation (SD) when normally distributed, or as median and interquartile range (IQR) in the case of non-normal distribution. Categorical data were reported as frequencies and percentages.

To compare two groups—such as in the case of partial nephrectomy procedures performed using the Da Vinci and Hugo™ platforms—the Student’s t-test was applied when the data followed a normal distribution, while the Mann-Whitney U test was used for non-normally distributed variables. For categorical variables, differences between groups were evaluated using the Chi-square test.

When the comparison involved three groups—as in the case of radical prostatectomy performed with the Da Vinci, Hugo™, and Versius^®^ systems—data were analysed using one-way ANOVA for normally distributed variables, and the Kruskal-Wallis test for non-normally distributed variables. In instances where significant differences emerged among the three groups, Dunn’s post hoc test was applied to identify which specific groups were statistically different.

All statistical tests were two-tailed, and a p-value ≤ 0.05 was considered indicative of statistical significance.

## Results

In this section, the clinical and economic outcomes of robotic-assisted surgery for two specific urological procedures are presented: partial nephrectomy (paragraph 3.1) and radical prostatectomy (paragraph 3.2). The results are reported for the three robotic platforms under evaluation: the Da Vinci, Hugo™ RAS, and Versius^®^ systems. For each procedure, data on patient demographics, operative times, costs, hospital stay, and post-operative complications are provided. The comparative analysis between the different robotic platforms aims to highlight both the clinical and economic implications of using each system in the context of urologic surgery.

### Partial nephrectomy

A total of 77 robotic-assisted partial nephrectomies were analysed: 57 performed with the Da Vinci Surgical System and 20 with the Hugo™ RAS. The comparison is reported in Table 1.

The two cohorts were comparable in terms of patient demographics. Mean patient age was 64.51 ± 10.57 years in the Da Vinci group and 66.4 ± 9.17 years in the Hugo™’s group, with no statistically significant difference (*p* = 0.48). The gender distribution was also similar between groups (*p* = 0.49), with a slight male predominance in both (57.9% and 70%, respectively).

Postoperative complications were rare and limited to two cases in the Da Vinci group, with none reported in the Hugo™’s group. The mean operative time was slightly longer in the Hugo™’s cohort (226.1 ± 73.58 min) compared to Da Vinci (202.05 ± 78.41 min), although the difference was not statistically significant (*p* = 0.16).

However, a significant difference was observed in total operating room occupancy time, which was longer for the Hugo™ platform (338.1 ± 77.62 min) versus Da Vinci (299.86 ± 81.30 min) (*p* = 0.03). This difference contributed to higher mean operating room costs in the Hugo™’s group (€2979.11) compared to the Da Vinci group (€2652.56), which was also statistically significant (*p* = 0.04).

No significant differences were found in length of hospital stay (4.35 vs. 4.32 days, *p* = 0.71) or in the corresponding hospitalization costs (€1508.25 vs. €1433.42, *p* = 0.70).

Regarding robotic system-related costs, per-procedure equipment cost was slightly higher for Hugo™ (€1744) compared to Da Vinci (€1680), while the robotic kit cost was lower for Hugo™ (€1995) than Da Vinci (€2090.91).

The mean total cost per procedure, combining robotic, operative, and hospitalization components, was higher in the Hugo™’s group (€8226.36 ± 1481.85) than in the Da Vinci group (€7856.89 ± 1335.77), although the difference was not statistically significant (*p* = 0.16).

**Table 1:** Clinical and economic outcomes of robotic-assisted partial nephrectomy by surgical platform

Variable	Da Vinci (*n* = 57)	Hugo™ RAS (*n* = 20)	*p*-value
Age (years), mean (SD)	64.51 (10.57)	66.4 (9.17)	0.48
Gender, n (%)			0.49
Male	33 (57.9%)	14 (70%)	
Female	24 (42.1%)	6 (30%)	
Post-operative complications, n	2	0	–
Operative time (min), mean (SD)	202.05 (78.41)	226.1 (73.6)	0.16
OR time (min), mean (SD)	299.86 (81.30)	338.1 (77.62)	**0.03**
OR cost (€), mean (SD)	2652.56 (805.72)	2979.11 (764.41)	**0.04**
Hospital stay (days), mean (SD)	4.32 (2.39)	4.35 (1.98)	0.71
Hospitalization cost (€), mean (SD)	1433.42 (814.21)	1508.25 (859.36)	0.70
Equipment cost (€)	1680	1744	–
Robotic kit cost (€)	2090.91	1995	–
Total cost (€), mean (SD)	7856.89 (1335.77)	8226.36 (1481.85)	0.16

OR = Operating Room. Total cost includes equipment, kit, OR, and hospitalization costs. Values are reported as mean (standard deviation) unless otherwise specified

### Radical prostatectomy

A total of 284 robotic-assisted radical prostatectomies were analysed, performed with three different platforms: **Da Vinci** (*n* = 200), **Hugo™ RAS** (*n* = 77), and **Versius**^®^ (*n* = 7). Table 2 provides a summary of the different variable analysed.

**Table 2:** Clinical and economic outcomes of robotic-assisted radical prostatectomy by surgical platform

Variable	Da Vinci (*n* = 200)	Hugo™ RAS (*n* = 77)	Versius^®^ (*n* = 7)	*p*-value
Age (years), mean (SD)	66.53 (7.26)	67.10 (5.88)	62.57 (6.85)	0.29
Post-operative complications, n	1	0	0	–
Operative time (min), mean (SD)	286.23(72.33)	302.74 (64.75)	370.57 (43.56)	**0.0046**
OR time (min), mean (SD)	319.88 (77.38)	338.96 (65.77)	417.14 (59.93)	**0.0025**
OR cost (€), mean (SD)	3003.88 (804.90)	3117.64 (702.33)	3751.77 (618.15)	**0.038**
Hospital stay (days), mean (SD)	3.40 (2.22)	3.01 (1.54)	2.86 (0.90)	0.13
Hospitalization cost (€), mean (SD)	1147.79 (851.57)	1001.28 (527.97)	940.0 (296.01)	0.12
Equipment cost (€)	1680	1744	1738.2	–
Robotic kit cost (€)	2246.3	1995	1995	–
Total cost (€), mean (SD)	8077.99 (1340.36)	7867.92 (996.76)	8430.76 (686.65)	0.13

OR = Operating Room. Total cost includes equipment, kit, OR, and hospitalization costs. Values are reported as mean (standard deviation) unless otherwise specified

The mean age of patients was comparable across the groups, with no statistically significant differences observed (*p* = 0.29). The overall incidence of post-operative complications was extremely low, with only one complication reported in the Da Vinci group and none in the Hugo™ and Versius^®^ cohorts.

The mean operative time was significantly different among the platforms (*p* = 0.0046), with the Versius^®^ group showing the longest mean duration (370.57 ± 43.56 min), followed by Hugo™ (302.74 ± 64.75 min) and Da Vinci (286.23 ± 72.33 min). Similarly, the total operating room occupancy time (including setup and closure) differed significantly (*p* = 0.0025), with Versius^®^ again presenting the highest value (417.14 ± 59.93 min), compared to Hugo™ (338.96 ± 65.77 min) and Da Vinci (319.88 ± 77.38 min).

These differences in operative and room times were reflected in the mean operating room costs, which were significantly higher for Versius^®^ (€3751.77 ± 618.15) compared to Hugo™ (€3117.64 ± 702.33) and Da Vinci (€3003.88 ± 804.90) (*p* = 0.038).

No statistically significant differences were observed in length of stay (*p* = 0.13), with all groups showing similar hospitalization durations. This was also reflected in the mean hospitalization costs, which were lowest in the Versius^®^ group (€940 ± 296.01) and highest in the Da Vinci group (€1147.79 ± 851.57), although the difference was not statistically significant (*p* = 0.12).

Regarding robotic system-related costs, per-procedure values were calculated based on regional procurement agreements, resulting in slightly higher equipment costs for Hugo™ (€1744) and Versius^®^ (€1738.2) compared to Da Vinci (€1680). Similarly, the robotic kit costs were lower for Hugo™ and Versius^®^ (€1995 each) compared to Da Vinci (€2246.3).

The total mean cost per procedure (including robotic, operative, and hospitalization costs) was highest in the Versius^®^’s group (€8430.76), followed by Hugo™ (€7867.92) and Da Vinci (€8077.99), but without reaching statistical significance (*p* = 0.09).

### Sensitivity analysis

Sensitivity analysis showed that total direct costs were sensitive to annual procedural volume through changes in equipment cost allocation. Lower annual utilization increased total direct costs across all platforms, while higher utilization reduced them. However, the relative differences between platforms remained limited across the explored scenarios, and the overall interpretation of broadly comparable direct costs within this institutional setting was unchanged. The results are reported in Tables 3, 4 and 5.

**Table 3:** Sensitivity analysis of equipment costs according to annual procedural volume

Annual volume	Da Vinci equipment €/case	Hugo™ equipment €/case	Versius^®^ equipment €/case
150	2800	2906,666667	2897,066667
200	2100	2180	2172,8
250	1680	1744	1738,24
300	1400	1453,333333	1448,533333
400	1050	1090	1086,4

**Table 4:** Sensitivity analysis of total direct costs for robotic-assisted radical prostatectomy according to annual procedural volume

Radical Prostatectomy				
Volume	Da Vinci	Hugo™	Versius^®^	Max Difference
150	9197,94	9020,586667	9583,836667	563,25
200	8497,94	8293,92	8859,57	565,65
250	8077,94	7857,92	8425,01	567,09
300	7797,94	7567,253333	8135,303333	568,05
400	7447,94	7203,92	7773,17	569,25

**Table 5:** Sensitivity analysis of total direct costs for robotic-assisted partial nephrectomy according to annual procedural volume

Partial Nephrectomy			
Volume	Da Vinci	Hugo™	Max Difference
150	8976,886257	9389,02465	412,1383927
200	8276,886257	8662,357983	385,471726
250	7856,886257	8226,357983	369,471726
300	7576,886257	7935,691317	358,8050594
400	7226,886257	7572,357983	345,471726

## Discussion

This study aimed to compare the direct costs associated with robotic-assisted surgery performed using the Da Vinci, Hugo™ RAS, and Versius^®^ platforms. However, the three-platform comparison was limited to radical prostatectomy, whereas partial nephrectomy involved only Da Vinci and Hugo™ systems. Overall, no statistically significant differences in total direct costs were observed among the evaluated platforms. However, the limited number of Versius^®^ procedures substantially restricts the strength of comparative inference. Although Versius^®^ demonstrated longer operative times, total direct costs did not significantly differ across platforms; however, this finding should be interpreted cautiously because of the small Versius^®^ cohort. This difference in operative duration could be attributed to the relatively low number of procedures performed with Versius^®^, which likely impacted the statistical significance of these results. Additionally, it is possible that surgeons are more experienced with the Da Vinci and Hugo™ systems, contributing to shorter operative times.

Despite these variations in the duration of surgery, the total procedure costs—including those associated with the robotic systems, kits, operating room use, and hospitalization—did not differ significantly. These findings suggest that direct procedural costs may be broadly comparable within our institutional setting; however, the small Versius^®^ cohort limits the robustness of this observation.

Interestingly, a similar study that focused on the comparison of Hugo™ and Da Vinci systems found comparable results regarding total costs [16]. However, in that study, the operative times for both platforms were shorter, leading to reduced operating room costs compared to our study. The differences in the operative time between our study and this comparison study could be explained by the differing experience levels of surgeons and the fact that the number of procedures with Versius^®^ in our study was relatively low, which might have skewed the results. In this way, the longer operative time with Versius^®^ in our study led to a higher operating room cost, which was reflected in the results. However, despite these differences, the overall cost structure remained similar across all three platforms in both studies, suggesting that while there may be small fluctuations, the total cost burden is comparable.

From a clinical perspective, this study adds valuable evidence to the ongoing discussion about the economic feasibility of robotic surgery. As the use of robotic systems continues to expand in clinical practice, understanding the cost structure of these systems becomes crucial for institutions considering their adoption. This study suggests that, should clinical outcomes be comparable across platforms—an assertion supported by previous research [10–15]—there may be a true competitive market emerging in the robotic surgery segment. Increased competition could drive down costs and encourage technological advancements, which would ultimately benefit both healthcare providers and patients. These findings could be instrumental for healthcare institutions when making strategic decisions about the acquisition of robotic surgery systems, especially when budget constraints are a significant consideration.

Our findings are generally consistent with previous studies evaluating the adoption of emerging robotic platforms in urologic surgery [19]. Recent reports on the Hugo™ RAS system have demonstrated the feasibility and safety of robot-assisted radical prostatectomy and partial nephrectomy, with perioperative outcomes broadly comparable to established robotic approaches [20]. Furthermore, comparative studies highlighted that differences in operative times are frequently influenced by institutional experience, workflow organization, and the progressive learning curve associated with newly introduced robotic systems [17, 18, 22].

However, several limitations must be acknowledged. First, detailed clinical and oncological variables potentially influencing operative complexity—such as BMI, prostate volume, lymph node dissection, nerve-sparing status, tumour complexity scores, ischemia time, blood loss, and patient comorbidities—were not consistently available and therefore could not be included in the analysis. Consequently, residual differences in case complexity between groups cannot be excluded, which may have influenced operative times and direct costs. Additionally, the study’s data set was unbalanced, with a significantly higher number of procedures performed using the Da Vinci system compared to Hugo™ and Versius^®^, which could have skewed the results. The analysis did not include readmissions, post-discharge complications, reinterventions, rehabilitation, or other extended-care costs. This is another limitation that may have affected the total cost calculation. Therefore, the reported estimates reflect only direct in-hospital expenditure. Moreover, this study focused exclusively on urological procedures, and while robotic surgery is widely used in this specialty, further research including other surgical disciplines would be valuable to verify whether these findings hold true across various types of surgery. These findings should be interpreted carefully. The Versius^®^ cohort included only seven procedures, making comparative statistical analyses inherently fragile and potentially influenced by case selection, surgeon adaptation, and residual learning-curve effects. This was partially related to the later clinical introduction of the platform in the urology department during the study period, resulting in fewer eligible procedures available for analysis.

The sensitivity analysis confirmed that annual procedural volume is an important determinant of robotic equipment cost allocation. Although total direct costs varied according to utilization rates, the relative differences between platforms remained limited across the explored scenarios. Nevertheless, the economic sustainability of robotic platforms remains strongly dependent on local surgical volumes and procurement agreements, which may limit the generalizability of the present findings.

In conclusion, while there are some differences in the duration of surgery and the associated operating room costs between the Da Vinci, Hugo™, and Versius^®^ systems, the overall economic costs of robotic-assisted prostatectomy and nephrectomy procedures are similar. In this single-center experience, direct procedural costs appeared broadly comparable across platforms, although conclusions regarding Versius^®^ remain exploratory because of the limited sample size. This study provides essential insights for healthcare providers, informing decisions about robotic surgery adoption and demonstrating that, with appropriate evidence, robotic surgery systems can be assessed not only for their clinical efficacy but also for their economic impact. However, these findings should be interpreted cautiously due to the retrospective design, unbalanced sample sizes, absence of detailed case-mix adjustment, and the limited number of Versius^®^ procedures. The present study should therefore be considered exploratory and hypothesis-generating rather than definitive. Further multicenter studies with larger cohorts and more comprehensive clinical and economic endpoints are needed to better define the comparative economic impact of emerging robotic platforms.

## Data Availability

The data supporting the conclusions of this article are available from ASST Grande Ospedale Metropolitano Niguarda, but they are subject to restrictions, as they were accessed under license for the purposes of this research and so are not publicly available. The data are, however, available from the authors upon reasonable request and with the permission of ASST Grande Ospedale Metropolitano Niguarda.
